# *Cyphellophora* sp. Isolated from a Corneal Ulcer in the Human Eye

**DOI:** 10.1155/2020/5861258

**Published:** 2020-07-22

**Authors:** Satheitra Rajandran, Kursiah Mohd Razali, Mushawiahti Mustapha, Prem Ananth Palaniappan, Fairuz Amran

**Affiliations:** ^1^Department of Ophthalmology, Hospital Raja Permaisuri Bainun, Jalan Raja Ashman Shah, 30450 Ipoh, Perak, Malaysia; ^2^Department of Ophthalmology, Hospital Canselor Tuanku Muhriz, Jalan Yaacob Latif, Bandar Tun Razak, 56000 Cheras, Kuala Lumpur, Malaysia; ^3^Institute for Medical Research, Jalan Pahang, 50588 Kuala Lumpur, Wilayah Persekutuan Kuala Lumpur, Malaysia

## Abstract

*Cyphellophora* is a black yeast-like fungus with most of the strains being isolated from soil and plants. It tends to cause sooty blotch and flyspeck disease in plants. In humans, it is known to cause superficial skin and nail infections. This report highlights the case of a patient who initially presented with a small corneal abrasion which rapidly progressed into a corneal ulcer after the patient did not respond to the initial conventional treatment. The laboratory results from the corneal scraping found it to be *Cyphellophora* sp.

## 1. Introduction

A corneal ulcer is an inflammatory or infective condition of the cornea resulting in the disruption of the epithelial layer and the corneal stroma [1]. The incidence of corneal ulcers has been estimated to occur 1.5–2 million times per year in developing countries [2]. The etiological agents of the microbial keratitis can be bacteria, fungi, or parasites [1, 2].

Fungal corneal ulcers usually occur in young workers involved in outdoor work, often agricultural work. Often, the fungus that causes a corneal ulcer belongs to the yeast group, e.g., *Candida* sp., or filamentous fungi, e.g., *Fusarium* and *Aspergillus*. A common predisposing factor is trauma to the eye due to organic matter or a soil-contaminated object [3].

*Cyphellophora* belongs to the genus *Cyphellophoraceae* and the order *Chaetothyriales*. It is a black, yeast-like fungus characterised by the production of septated conidia from intercalary or terminal phialides which bear flaring, thin, or conspicuous collarettes. The first generic species *Cyphellophora laciniata* was discovered and isolated from the human skin in 1962 [4, 5].

Plant-associated *Cyphellophora* species such as *C. guyanesis* and *C. vermispora* were commonly found in soil and plants. Most of the strains were known to cause sooty blotch and flyspeck disease which are known to damage certain fruit crops [4].

We describe a rare case of *Cyphellophora* sp. isolated from a patient with a corneal ulcer. This patient initially presented with a corneal abrasion; however, it progressed rapidly into a corneal ulcer. The ulcer did not have the typical presentation of fungal infection and did not respond well to the available topical antifungal eye drops. Here, we highlight the difficulty and the challenges both in the clinical diagnosis and the treatment of this patient.

## 2. Case Report

A 58-year-old male with poorly controlled diabetes mellitus who works as a security guard presented with discomfort and redness in the left eye after a foreign body entered his eye while riding a motorbike. He immediately rinsed out his eye with tap water. On presentation, it was noted that the left eye vision was 6/36 and the pinhole was 6/18. Examination of the left eye showed a small foreign body (<1 mm) located centrally on the cornea at 12 o'clock at the level of superior pupillary margin. The foreign body was removed, leaving a small epithelial defect. The patient was discharged and prescribed with topical chloramphenicol 0.5% every 4hourly. However, during the subsequent review, it was noted that the epithelial defect persisted.

Ten days later, the patient presented with worsening eye pain and redness. The vision of the left eye was 6/24, and an examination showed a stromal infiltrate measuring 1.7 mm (vertically) and 1.8 mm (horizontally) along with Descemet folds and surrounding corneal haziness. There were no signs of fluffy edges, satellite lesions, or endothelial plaque, which would suggest a fungal infection. Initially, the patient was treated as having infective bacterial keratitis and given hourly topical gentamicin 0.9% and ceftazidime 5%. A corneal scraping was sent for analysis.

**Figure 1:**
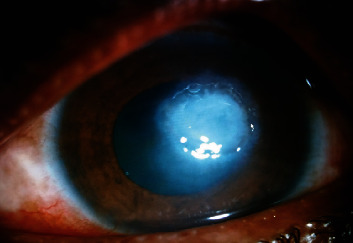
Anterior segment picture of the left eye showing a corneal ulcer involving the visual axis.

Despite intensive hourly topical antibacterial inpatient treatment, the infection progressed into a large corneal ulcer affecting the central visual axis (Figure 1). The visual acuity dropped to 1/60.

The initial lab results of the corneal scraping showed a fungal isolate; however, it had to be sent to the Institute for Medical Research Malaysia (IMR) for identification in view of it being a new strain. The patient was started on hourly topical Amphotericin B 0.15% and hourly Fluconazole 0.2% along with oral Fluconazole 200 mg once daily in addition to the topical treatment. As his diabetic control was very poor, the patient was referred to a medical team and dietitian for optimisation of his diabetes.

Three weeks from the first presentation, the patient was given an intrastromal injection of Amphotericin B (5 *μ*g in 0.1 ml). A week later, he underwent an intracameral administration of 5 *μ*g in 0.1 ml Amphotericin B as he was not responding well to the topical and oral treatments.

**Figure 2:**
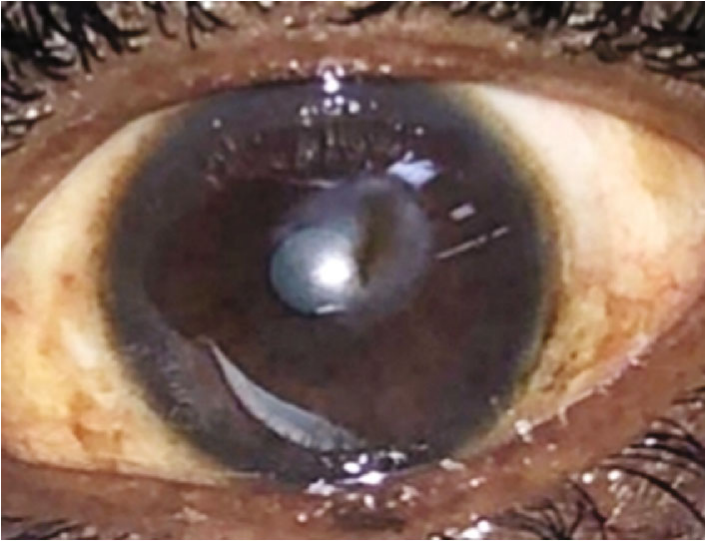
Corneal scarring centrally (6 months after the initial presentation).

Initially, the patient did not show good response to the intrastromal and intracameral injections. However, a week later, the ulcer started responding in addition to the combination of intensive topical antifungal (hourly Fluconazole 0.2% and Amphotericin B 0.15%) and antibacterial (2 hourly gentamicin 0.9% and cefuroxime 5%) eye drops. Four months later, the ulcer had healed completely, leaving a corneal scar (Figure 2). His best corrected visual acuity was 6/24.

**Figure 3:**
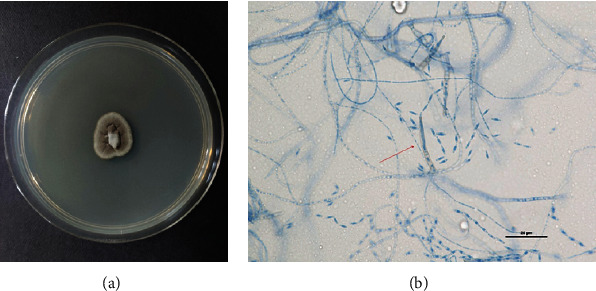
(a) *Cyphellophora* sp. colony growth on Sabouraud dextrose agar. (b) LPCB mount: phialides with collarettes bearing septated conidia (magnification 40x).

The final lab results from IMR suggested an isolate of fungus from *Cyphellophora* sp. The method that was used for microscopic identification of this fungus was culturing on Sabouraud dextrose agar (Figure 3(a)). Lactophenol cotton blue mounting revealed phialides with collarettes bearing septated conidia (Figure 3(b)).

## 3. Discussion

According to the literature, there are only a few reports on *Cyphellophora* causing superficial cutaneous and nail infections in humans. *Cyphellophora laciniata*, *C. pluriseptata*, and *P. ambigua* have been reported to be isolated from superficial lesions in human cutaneous infections [6, 7]. In another instance, the bronchoalveolar lavage fluid from a patient after heart bypass surgery grew *C. fusaroides* [8]. *Cyphellophora suttonii* was also isolated from an ulcerating skin lesion in a patient with sarcoidosis [9].

This was a rare instance of this fungus isolated from an ulcer in the eye. We faced difficulties in the preliminary diagnosis as it did not have typical characteristics of a fungal infection in the eye. It showed a rapid progression of the infection despite being on antibacterial eye drops, which could have also been exacerbated by the patient's poor control of his diabetes. The treatment was challenging as it did not show a good response to any of the initial approaches. Fortunately, the laboratory results aided in the treatment revision. A combination of intensive antifungal and antibacterial eye drops hastened the recovery and healing process, which could suggest a possibility of a mixed infection.

In vitro study analysis of the susceptibility of the fungus *Cyphellophora* sp. showed a good response to caspofungin (minimum inhibitory concentration (MIC) 1.1 *μ*g/ml) and newer types of azoles such as itraconazole and voriconazole (0.1 *μ*g/ml and 0.3 *μ*g/ml, respectively). In the study, it was also noted that the MIC was the highest in susceptibility testing of Fluconazole (27.6 *μ*g/ml) and Amphotericin B (4 *μ*g/ml). This could explain the initial poor response to the treatment in this patient [10]. However, there are no in vivo studies or other similar clinical case reports that could aid in the management.

## 4. Conclusion

The *Cyphellophora* fungus has now been isolated from a corneal ulcer in a human. The exact nature of the infection and the susceptibility to the antifungal treatment are yet to be learned as this was the first incidence of this fungus in a corneal ulcer in the human eye.

## Data Availability

The data that support the findings of this study are available from the corresponding author upon reasonable request.
